# Fast Detection of Cadmium in Chocolate by Solid Sampling Electrothermal Vaporization Atomic Absorption Spectrometry and Its Application on Dietary Exposure Risk Assessment

**DOI:** 10.3390/molecules27196197

**Published:** 2022-09-21

**Authors:** Hongyu Jia, Xue Li, Guanyu Lan, Zhaohui Wang, Li Feng, Xuefei Mao

**Affiliations:** 1 Key Lab of National Soybean Industry Technology System, School of Food Science and Engineering, Jilin Agricultural University, Changchun 130118, China; 2 Institute of Quality Standard & Testing Technology for Agro-Products, Chinese Academy of Agricultural Sciences, Key Laboratory of Agro-Food Safety and Quality, Ministry of Agriculture and Rural Affairs, Beijing 100081, China; 3 Research and Development Department, Changsha Kaiyuan Hongsheng Technology Co., Ltd., Changsha 410199, China

**Keywords:** cadmium, electrothermal vaporization atomic absorption spectrometry, solid sampling, chocolate, dietary exposure risk assessment

## Abstract

In this work, a rapid detection method using solid sampling electrothermal vaporization atomic absorption spectrometry (SS-ETV-AAS) was established for cadmium in chocolate. The instrumental system includes a solid sampling ETV unit, a catalytic pyrolysis furnace, an AAS detector, and a gas supply system with only an air pump and a hydrogen generator. Herein, MgO material with 1.0–1.5 mm particle size was first employed to replace the kaolin filler previously used to further shorten the peak width and to thereby improve the sensitivity. With 350 mL/min of air, a chocolate sample was heated for 25 s from 435 to 464 °C to remove water and organic matrices; then, after supplying 240 mL/min hydrogen and turning down air to 120 mL/min, a N_2_/H_2_ mixture gas was formed to accelerate Cd vaporization from chocolate residue under 465 to 765 °C. Under the optimized conditions, the detection limit (LOD) was obviously lowered to 70 pg/g (vs. previous 150 pg/g) with R^2^ > 0.999; the relative standard deviations (RSD) of repeated measurements for real chocolate samples ranged from 1.5% to 6.4%, indicating a favorable precision; and the Cd recoveries were in the range of 93–107%, proving a satisfied accuracy. Thus, the total analysis time is less than 3 min without the sample digestion process. Thereafter, 78 chocolate samples with different brands from 9 producing countries in China market were collected and measured by this proposed method. Based on the measured Cd concentrations, a dietary exposure assessment was performed for Chinese residents, and the target hazard quotient (THQ) values are all less than 1, proving no significant health risk from intaking chocolate cadmium for Chinese residents.

## 1. Introduction

Chocolate, as a popular snack reaching USD 98 billion in sales in 2021 all over the world [1], is mainly made from the cocoa bean and other components or additives. In recent years, with the rapid development of China’s market, chocolate products have become one of the favorite foods, especially for Chinese teenagers [2]. However, cocoa raw materials are mainly produced from developing countries, such as Ghana [3], Ecuador [4], and Cote d’Ivoire [5], where they suffer from environmental contamination of heavy metals deriving from industrial or agricultural activities. Cadmium (Cd), as one of the Group I carcinogens by the International Agency for Research on Cancer (IARC), might result in renal tubular damage [6], renal failure [7], bone disease [8], and breast [9], prostate [10], lung [11], and pancreatic cancer risks [12] via dietary intake. So, the Joint Expert Committee on International Food Additives (JECFA) has reset the tolerable monthly intake of Cd to 25 μg/kg body weight (bw) [13]. In 2021, the Codex Alimentarius Commission (CAC) revised the maximum limits of Cd in chocolate as 0.3 mg/kg for containing ≤ 30% cocoa and 0.7 mg/kg for 30–50%, respectively [14]. Peixoto et al. investigated the daily consumption of chocolate beverage powder for Brazilian children and found that 4–35% of the weekly tolerable temporary intake of Cd could be contributed to chocolate-related dietary exposure [15]. In China, Cd contamination in rice is also a concerning problem of food safety [16]; as well, the average monthly dietary exposure of Cd to Chinese residents is 9.9 μg/kg bw, while southern Chinese might be confronted with a higher Cd exposure level [17]. Thus, Chinese residents must reduce Cd intake from other food sources as much as possible. To the best of our knowledge, dietary exposure assessment of Cd from chocolate consumption for Chinese has not been reported so far. Further, rapid and precise analysis of Cd is also indispensable to monitor the actual presence of chocolate Cd prior to risk assessment.

To detect Cd in food samples, graphite furnace atomic absorption spectrometry [18] (GF-AAS), inductively coupled plasma mass spectrometry [19] (ICP-MS), hydride generation atomic fluorescence spectrometry [20] (HG-AFS), anodic stripping voltammetry [21] (ASV), etc., have been commonly employed as laboratorial approaches. These above methods demonstrate high sensitivity and good accuracy; however, they are complicated, have high power consumption, and fail to render the rapid detection of Cd in food samples due to acid digestion and liquid sampling procedures regardless of toxic reagent consumption and environmental contamination. Additionally, chocolate is rich in cocoa butter, in which the lipid components are difficult in being completely digested via the regular digestion method [22]. Thus, it is necessary to establish a rapid analysis method of Cd in chocolate without the digestion process.

Electrothermal vaporization (ETV), as an excellent and versatile sampling approach, has been frequently used to couple with AFS [23], AAS [24], ICP-MS [25], or inductively coupled plasma optical emission spectrometry (ICP-OES). ETV enables solid sampling analysis with high sampling efficiency, small sample size, and short analysis time without toxic reagent consumption. In terms of the vaporization materials, ETV can be fabricated as strips, coils, rods, tubes, or furnaces made of high melting point metals or graphite. Among them, solid sampling graphite furnace AAS (SS-GF-AAS) has frequently been employed to detect Cd via direct solid sampling in grain samples [26]. However, the maximum sample size was <1 mg due to the GF’s structure and matrix effect. On the other hand, Bustos and Toro et al. [27] developed a high-resolution continuous source graphite furnace atomic absorption spectrometry (HR-CS-GF-AAS) method for simultaneous determination of Cd and Ni in slurry chocolate samples rather than direct solid sampling analysis. To increase the sampling size for solid aliquots, Mao and Feng et al. [28,29] fabricated a quartz tube wrapped with Ni-Cr heating coils as the ETV unit to vaporize Cd in max. 200 mg grain and tea samples. Herein, the pyrolysis furnace filled with kaolin is responsible for absorbing gaseous interferents and retaining the pre-vaporized Cd from the sample. Furthermore, a novel gas system consumed only air and water to orderly yield air and reducing (H_2_ and N_2_ mixture) atmospheres to facilitate sample ashing and Cd vaporization [29]. As a result, the method detection limit (LOD) reached 0.15 ng/g using a 200 mg grain sample. Due to the resistance and absorption of the kaolin filler, the peak width of the Cd signal is wider than that without the pyrolysis filler. The pyrolysis furnace filler should be modified to reduce the peak width, and then the analytical sensitivity can be improved further. Even so, if this solid sampling ETV-AAS method is applied to the detection of Cd in chocolate, the monitoring process will be easier, faster, and greener.

In this work, the kaolin filler of the ETV-AAS instrument was replaced with MgO particles, and the Cd peak width fulfilled a significant reduction. Then, the newly modified ETV-AAS instrumentation was fabricated, and the instrumental conditions were optimized. As a result, the absolute LOD of 70 pg/g Cd in chocolate was reached and is largely better than 150 pg/g by the previous apparatus. Thereafter, 78 chocolate samples with different brands from 9 producing countries from the Chinese market were collected and measured by this proposed method. Based on these data, the dietary exposure assessment of Cd in chocolate for Chinese residents was carried out. This work rendered a new analysis method of Cd in chocolate and helped us learn about the health risk of chocolate Cd for Chinese consumers.

## 2. Results and Discussion

### 2.1. Modification of the Pyrolysis Filler

**Figure 1:**
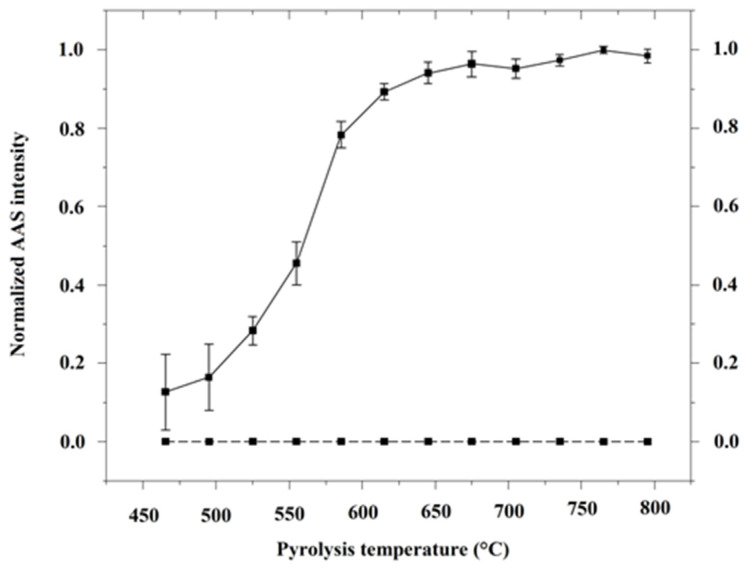
Cd signal peak comparison of MgO and Kaolin fillers using SS-ETV-AAS. The GBW(E)100718 CRMs of peanut powder had a Cd concentration of 62 ± 3 ng/g, and the sampling size was 0.1 g.

In a previous study [28], the pyrolysis furnace was filled with kaolin filler with 0.5–1.0 mm particle size, leading to a wider Cd peak than that without filler. This might be due to the resistance and absorption of kaolin filler. In fact, kaolin, MgO, and attapulgite clay are promising fillers based on limiting metal vaporization and reducing leachability under O_2_-containing atmosphere [30]. To reduce the peak width, the leachability of Cd through the pyrolysis filler must be enhanced, so the fillers’ particle size might be increased to enlarge the intervals among particles to accelerate the transportation of Cd analyte. Herein, MgO material with 1.0–1.5 mm particle size was accessible in our laboratory. So, we compared the Cd peak shapes between kaolin and MgO materials being filled in the pyrolysis furnace, and the results are shown in Figure 1. Obviously, the half peak width (1.669 s) of MgO is narrower by ~59% than that (4.102 s) of kaolin with the same peak area (0.98:1) possibly due to fewer micro pores in MgO particle materials, agreeing with our premise as well as proving a better analysis sensitivity. As a result, MgO was chosen as the optimized pyrolysis filler.

### 2.2. Sample Dehydration and Cd Pre-Vaporization

**Figure 2:**
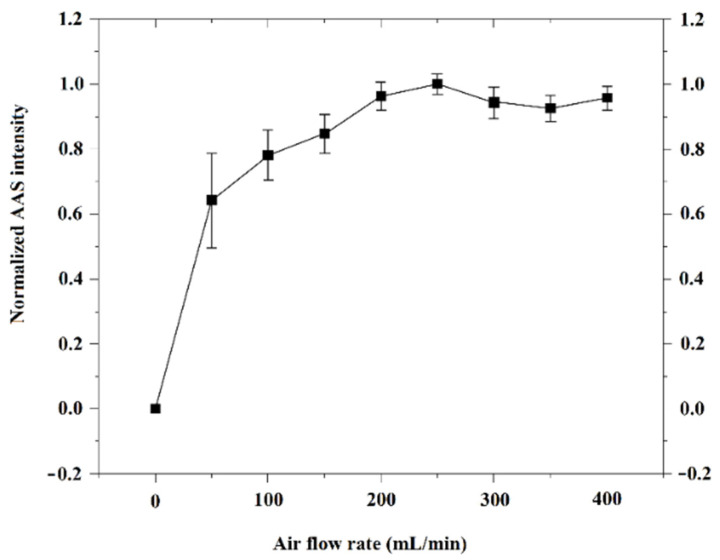
Effect of pre-vaporization temperature on Cd signal. The Cd signal intensity under 765 °C was set as 1, and the others were normalized with it. Solid line: N_2_/H_2_ mixture atmosphere was employed; dotted line: N_2_/H_2_ mixture atmosphere was invalid.

Chocolate samples are rich in cocoa butter, carbohydrates, proteins, minerals, etc. So, before Cd introduction, moisture and organic substances must be removed from the sample matrix to avoid the following interference as much as possible. According to a previous study [28], Cd in grain samples was completely ashed before 765 °C, so 435–464 °C heating was chosen in this work to remove moisture and organic substances in the chocolate sample, and then 465–765 °C heating was continued to ash the sample and pre-vaporize Cd analyte from residue. To investigate the efficacy of dehydration and pre-vaporization, a peanut powder CRM (GBW(E)100718) was loaded into the ETV unit and heated according to the procedures mentioned above. When heating to 464 °C (~25 s), the sample was completely dehydrated and started to be partially ashed via visual inspection, and the pictures are shown in Figure S1. After heating to 765 °C (~85 s), the AAS did not measure any Cd signal in Figure 2, proving no breakthrough of Cd into the detector. This is in accordance with the previous result [22] that a small fraction of Cd analyte was pre-vaporized and then trapped by the pyrolysis filler. As shown in Figure 2, the Cd signals under more than 700 °C indicate a plateau with 4–7% RSDs, proving that MgO filler also fulfills the retention of re-vaporized Cd for the following vaporization and release processes under a N_2_/H_2_ mixture atmosphere. Therefore, heating to 765 °C was selected as the sample dehydration and Cd pre-vaporization condition.

### 2.3. Gas Atmosphere and Condition

**Figure 3:**
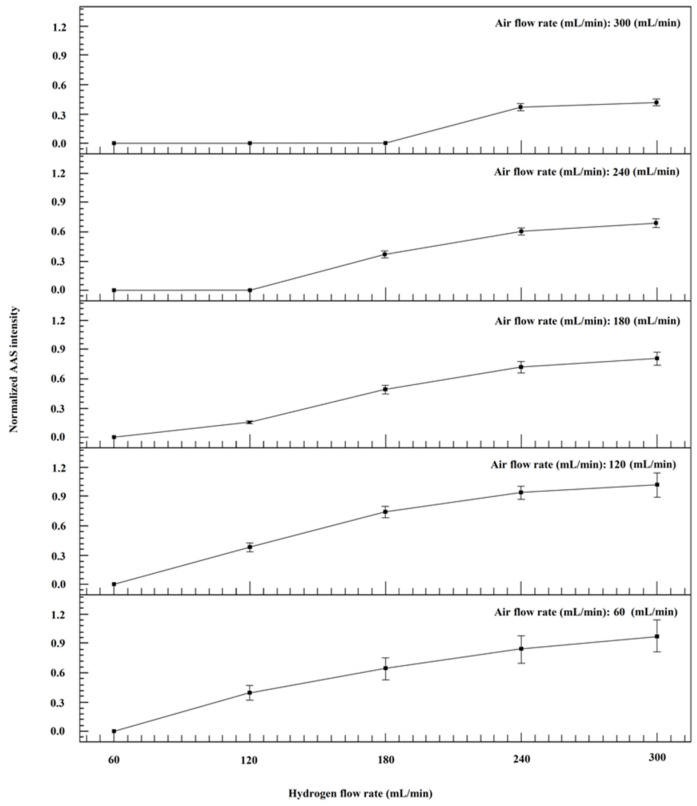
Optimization of air flow rate during the dehydration and pre-vaporization procedures. Herein, the CRM of peanut powder (GBW(E)100718) was measured by the ETV-AAS using different air flow rates. Other experimental conditions were performed as shown in Table 1. The Cd intensity under 250 mL/min air flow rate was set as 1, and others were normalized with it.

According to a previous study, an O_2_-rich atmosphere facilitates cadmium to form oxides, which are difficult to be vaporized from residue vs. that under a reducing atmosphere. At this time, carrier air under high temperature (heating to 765 °C ut infra) is able to remove water and organic substances from the sample matrix, as well as to minimize the pre-vaporization of Cd as much as possible. Herein, the air flow rate during the dehydration and pre-vaporization procedures was investigated and the results are shown in Figure 3. Before 200 mL/min, the AAS intensity of Cd goes up with the increase of air flow rate, due to the completeness of sample ashing and matrix sweeping. As a result, 250 mL/min was chosen as the optimized air condition with the highest Cd intensity and smallest RSD for the following work.

**Figure 4:**
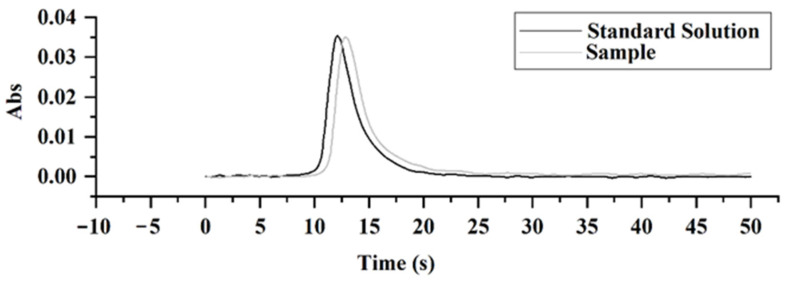
Optimization of H_2_ and air flow rates during the vaporization and detection procedures. Herein, the CRM of peanut powder (GBW(E)100718) was measured by the ETV-AAS using different H_2_ and air flow rates. Other experimental conditions were performed as shown in Table 1. The Cd intensity under 120 mL/min air + 300 mL/min H_2_ was set as 1, and others were normalized with it.

After sample ashing, the majority of Cd analyte was still kept in the residual ash. To enable the complete vaporization of Cd under the relatively lower temperature (~800 °C), the reducing atmosphere was indispensable to accelerate the atomization of Cd. According to a previous study [29], excess H_2_ consumes O_2_ in air via ignition to form a H_2_ and N_2_ mixture, getting rid of inconvenient and dangerous gas bottles. Considering the complicated matrix of chocolate, the gas atmosphere of H_2_ and air was investigated, and the results are shown in Figure 4. With the increment of H_2_ introduction, Cd intensities increase until 240 mL/min, possibly due to the atomization effect of H radicals, while the smaller flow rate of air results in the enhancement of Cd intensity until 120 mL/min. Finally, 120 mL/min air + 240 mL/min H_2_ with the favorable RSD (8.8%) was selected as the optimal condition.

### 2.4. Interference Study

**Figure 5:**
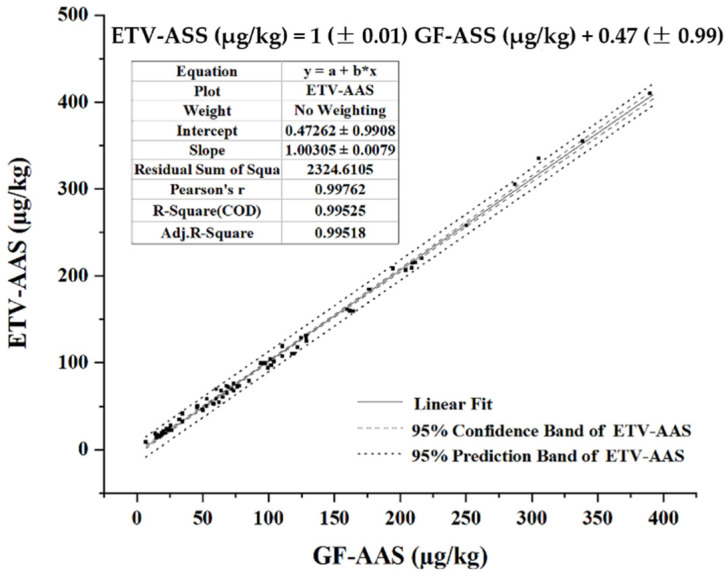
Comparison of Cd signal peaks between a real chocolate sample and standard solution. The Cd concentration of chocolate sample (No. 9) is 18.4 ± 2 ng/g; the Cd concentration of standard solution is 18 ng/mL.

To verify the anti-interference capability of this proposed method for the real sample, a chocolate sample was weighed by 100 mg to compare the Cd intensity using standard solutions with the same Cd mass. As shown in Figure 5, the chocolate peak is lightly delayed vs. the standard solution, possibly due to the complicated matrix of chocolate and a longer heating time for Cd vaporization. However, their peak shapes are almost consistent, and the peak area ratio of the chocolate to standard solution is 1:0.978, proving no obvious interference for Cd quantification. Therefore, the absence of interference made standard calibration possible for Cd detection.

### 2.5. Analytical Performance

The LOD was calculated to be 3 × SD/K based on the standard deviation (SD) and the slope of standard curve (K) of the blank solution measured 11 times, and the method LOD was 70 pg/g using a 0.1 g sample size. The linearity range of this method was 0.5–100 ng with R^2^ = 0.999. The RSD for the peanut powder CRM (GBW(E)100718) measured 11 times was 3.2%, indicating a favorable analytical precision.

**Figure 6:**
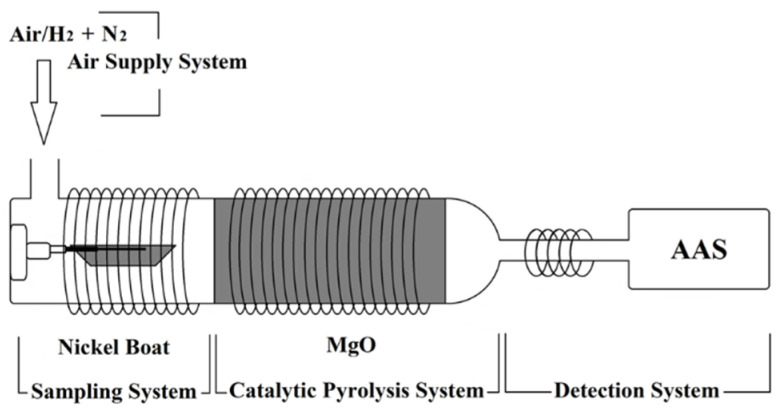
Comparison of Cd concentrations measured by the ETV-AAS and microwave digestion GF-AAS methods.

To verify the method feasibility, 78 chocolate samples from 9 countries (China, 7; United States, 9; Italy, 16; Germany, 13; Japan, 10; France, 5; Switzerland, 5; Russia, 9; Malaysia, 4) were collected from China’s market and then measured by the proposed method and the microwave digestion GF-AAS method, respectively. From Figure 6, the two resulting groups showed no significant difference (*p* > 0.05) with R^2^ = 0.9953, indicating a good accuracy and applicability. Meanwhile, the sample analysis time of this method can be controlled within 3 min without sample digestion, which can greatly improve the sample analysis efficiency. After replacing kaolin filler with MgO, the half width of the Cd signal peak was reduced by 59% and the absolute LOD of 70 pg/g was thereby lowered by 53% vs. 150 ng/g using kaolin filler in a previous study [29].

### 2.6. Cadmium Contamination of Chocolate in China Market and Its Dietary Exposure Risk Assessment

As shown in Table 2, the total average concentration of Cd in chocolate samples is 100.4 µg/kg. Among the original countries, the chocolate from Russia ranks first with an average of 225 µg/kg of cadmium; and the highest Cd concentration is 392 µg/kg, exceeding the maximum limit (0.3 mg/kg for containing ≤ 30% cocoa) of CAC. As well, the chocolate Cd from USA, China, and Malaysia exceeds 0.1 mg/kg. Herein, cocoa, as the most important raw material, readily accumulates Cd from soil and water [31]. Furthermore, cocoa products are mainly produced from developing countries in Africa and South America [32,33], where the contamination of heavy metals, including Cd, frequently affects food safety. However, from the Cd presence in chocolate in the Chinese market, the violation rate is very low according to the CAC standard.

Firstly, it was assumed that the daily chocolate intakes were 10, 50 and 100 g/d, respectively. According to Equations (1) and (2), the THQ results are shown in Table 3. In terms of the single intake route for chocolate, THQ_Average_ and other THQ values of chocolate Cd from 9 original countries sites are all < 1, proving no significant health risk from chocolate intake. Of course, the THQ values of Russia and USA are ≥0.3 (100 g/d intake), accounting for a remarkable ratio considering other Cd intake exposures. In fact, the Cd intake for Chinese residents is mainly derived from grain and vegetables and sometimes from animal food [13]. According to a previous study [13], the average monthly dietary exposure of Cd for Chinese residents is 9.9 μg/kg bw, while the average possible monthly dietary intake of cadmium in chocolate was 0.25 μg/kg bw, accounting for ~2.5% of the above, and the maximum possible monthly dietary intake was 0.98 μg/kg bw, accounting for 9.9% of the above. If integrating all Cd exposures from dietary intakes, the health risk of Cd for Chinese residents should be paid for more attention.

## 3. Materials and Methods

### 3.1. Instrumentation

**Figure 7:**
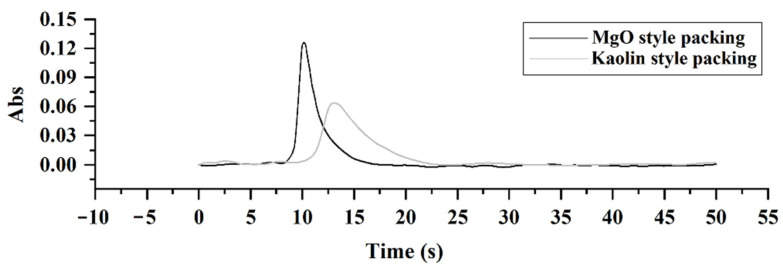
Structural diagram of SS-ETV-AAS instrumentation.

This SS-ETV-AAS instrumentation (Figure 7) was modified from the original ETV-AAS (AA2288, Kaiyuan Hocent Scientific Instrument, Changsha, China), which consists of a solid sampling ETV, a quartz pyrolysis furnace with an integrated outlet tube (IOT), a miniature AAS detector, and a gas supply system. The SS-ETV is mainly made of a quartz tube (Φ 19 mm) with electrothermal Ni-Cr coils to ash samples and vaporize Cd; as well, a nickel boat was selected for introducing a chocolate aliquot into ETV. The prolonged quartz tube of the ETV unit was also employed as the quartz pyrolysis furnace wrapped with electrothermal Ni-Cr coils; herein, the furnace was filled with kaolin or MgO fillers (vide infra) to absorb interferents and transport Cd analyte at 700–850 °C. The end of the quartz pyrolysis furnace was changed into a 2 mm diameter IOT wrapped by Ni-Cr coils to constrain the Cd atomization flame size; the miniature AAS mainly comprised a Cd hollow cathode lamp (HCL, 228.8 nm, Beijing Research Institute of Nonferrous Metals, Beijing, China), a quartz lens (Changsha Kaiyuan Hongsheng Technology, Changsha, China), and a photomultiplier tube (PMT). The gas supply system was connected to the ETV quartz tube, consisting of an air pump to provide O_2_, a hydrogen generator, an ignitor for burning H_2_ with O_2_ to yield N_2_/H_2_ mixture, and a water recycler. The detailed parameters of SS-ETV-AAS are shown in Table 1.

To verify the proposed method, a GF-AAS instrumentation (Model AA6800, Shimadzu, Kyoto, Japan) was utilized to measure Cd in chocolate samples coupled with a microwave digestion system (TOPEX, PreeKem Scientific Instrument, Shanghai, China). The detailed operating conditions of GF-AAS are shown in Table S1 and the specific operation of microwave digestion is shown in Table S2 (see Supplementary Materials).

### 3.2. Chemicals and Materials

Standard solution (1000 mg/L) of Cd and certified reference materials (CRMs) of peanut powder (GBW(E)100718, certified Cd value = 62 ± 3 ng/g) were purchased from the National Research Center for Certified Reference Materials of China (Beijing, China). Working standards were prepared by stepwise dilution of the stock standards with purified water from a Milli-Q Integral Water Purification System (Millipore, Burlington, MA, USA). Herein, 6 mLHNO_3_ and 2 mL H_2_O_2_ (guaranteed grade) were used for microwave digestion of 0.2 g chocolate samples. For GA-AAS, Pd(NO_3_)_2_ (analytical grade) was chosen as a chemical modifier; for the gas supply system, KOH (guaranteed grade) was employed to generate hydrogen with water electrolysis, all of which were purchased from Sinopharm Chemical Reagent (Beijing, China). MgO material was purchased from the Jiangsu Xianfeng Nanomaterial Technology Co., Ltd. (Nanjing, China).

For the method establishment and risk assessment, 78 chocolate samples with different brands from 9 producing countries (China, United States, Italy, Germany, Japan, Switzerland, New Zealand, Russia and Malaysia) were purchased from a Chinese market. After grinding at −2.6 °C and 28,000 rpm for 5 s using a low temperature mill (SM-3C, Xiangtai Precision Machinery Co., Ltd., Taiwan, China), chocolate samples were sieved by 0.178 mm mesh and then stored at 4 °C for further analysis.

### 3.3. Analytical Procedures of the SS-ETV-AAS Method

The SS-ETV-AAS analysis procedures are listed in Table 1 and can be categorized into four steps as follows: (1) Dehydration: The sample (0.1 g or less sample size) in the nickel sampling boat was first dried for 25 s from 435 to 464 °C under 350 mL/min air to fulfill dehydration and organic matrix elimination. (2) Pre-vaporization: Heating from 465 to 765 °C for 60 s was performed to reach the complete sample ashing, in which ~5% of Cd analyte was also vaporized and captured by the filler in the pyrolysis furnace. (3) Complete vaporization: When switching the gas into 120 mL/min air and 240 mL/min hydrogen, the N_2_/H_2_ mixture was formed through the combustion and condensation process; meanwhile, Cd analyte in the chocolate sample residue and that captured by filler were all vaporized and then transported into the IOT. (4) Detection: Vaporized Cd was atomized by the N_2_/H_2_ flames at the end of the IOT, and the HCL irradiated Cd atoms and AAS signals were acquired for Cd quantification via peak area. (5) Clean: After detection, the step (3) conditions were kept for 20 s to clean up the vaporization and transportation system, and then the carrier atmosphere was switched into 500 mL/min air and the temperature was decreased to 435 °C within 15 s.

### 3.4. Risk Assessment of Cd through Chocolate Consumption

The estimated daily intake (EDI, µg/kg/d) of Cd is related with the concentrations of Cd in the sample and chocolate consumption. The EDI was calculated with the following equation:

EDI = (E_F_ × E_D_ × F_IR_ × C)/(W_AB_ × T_A_) (1)

where E_F_ is the exposure frequency (365 days per year), E_D_ is the exposure duration (70 years), F_IR_ is the chocolate ingestion rate (g per person and day), C is the Cd concentration in chocolate (mg/kg), W_AB_ is the average body weight, and T_A_ is the average exposure time (E_F_ × E_D_). According to a previous study, F_IR_ was set as 4.975 g/d [2]; W_AB_ was selected as 60 kg bw in this work.

### 3.5. Target Hazard Quotient

Non-carcinogenic risks via consuming chocolate were assessed according to the target hazard quotient (THQ) (USEPA, 2000) as follows:

THQ = EDI/RfD(2)

where RfD is the reference dose of daily Cd intake, namely 1 µg/d/kg bw from the USEPA [34]. RfD is only used as a reference to measure the possibility of increasing the potential risk. When the exposure value gradually increases, the possibility of adverse health effects will increase. Even for groups exposed to RfD for life, it does not mean that will inevitably have an impact on their health. A THQ value < 1 indicates no significant risk for the human body; otherwise, it means an unacceptable risk. The higher the THQ, the greater the corresponding risk.

### 3.6. Statistical Analysis

Trial data were statistically analyzed using Origin 2022 and Microsoft Excel 2016 software.

## 4. Conclusions

In this work, a solid sampling ETV-AAS method of chocolate Cd was established without a sample digestion process. Herein, MgO was first employed as a pyrolysis filler to replace kaolin materials, and the Cd signal peak was thereby improved and the LOD of 70 pg/g was significantly lowered vs. 150 pg/g using a kaolin filler. Furthermore, the total analysis time was less than 3 min, which significantly accelerates the testing efficiency. Based on the measured Cd concentrations in 78 chocolate samples from 9 producing countries in China market, the THQ values are all less than 1, proving no significant health risk from chocolate cadmium intake for Chinese residents. The analysis and assessment process further verified the applicability and feasibility of this proposed ETV-AAS method, with advantages of rapidness, robustness, precision, and environmental friendliness.

## Figures and Tables

**Table 1:** Cd presence in chocolate samples measured by the ETV-AAS method (*n* = 3).

Countries	Producers	Minimum Value (µg/kg)	Maximum Value (µg/kg)	Average Value (µg/kg)	Standard Deviation (µg/kg)	Median Value (µg/kg)	Total Average (µg/kg)
CHN	7	52.8 ± 1.5	209.3 ± 3.1	135.4	62.1	128.6	100.4
USA	9	67.85 ± 0.9	253.2 ± 2.4	149.4	69.8	110.4	
ITA	16	15.1 ± 0.7	102.4 ± 1.3	44.2	28.4	40.1	
GER	13	6.7 ± 0.5	121.8 ± 2.2	48.6	36.9	32.3	
JPA	10	45.4 ± 1.4	146.2 ± 2.4	95.1	37.1	89.6	
CHE	5	34.6 ± 1.8	110.5 ± 0.8	69.9	31.7	60.2	
FRA	5	18.7 ± 0.9	99.5 ± 2.8	58.9	32.4	65.2	
RUS	9	94.5 ± 2.7	392.4 ± 4.2	225.1	109.7	159.8	
MAS	4	45.4 ± 0.6	173.4 ± 2.5	132.7	75.4	136.8	

**Table 2:** Health risk assessment of chocolate Cd intake for Chinese residents.

Countries	Average Cd (µg/kg)	THQ_10(g/d)_	THQ_50(g/d)_	THQ_100(g/d)_
CHN	135.4	0.027	0.136	0.272
USA	149.3	0.030	0.150	0.300
ITA	45.4	0.009	0.046	0.091
GER	50.5	0.010	0.051	0.101
JPA	95.1	0.019	0.095	0.191
CHE	69.8	0.014	0.070	0.140
FRA	58.9	0.012	0.059	0.118
RUS	225.1	0.038	0.188	0.375
MAS	132.7	0.022	0.111	0.221

**Table 3:** Operating parameters of the SS-ETV-AAS.

Program	Time (s)	Temperature (°C)	Carrier Gas	Flow Rate (mL/min)
Dehydration	25	435–464	Air	350
Pre-vaporization	60	465–765		
Complete vaporization and detection	50	765–767	N_2_/H_2_	Air: 120 H_2_: 240
Clean	20	767		
	15	767–435	Air	500

The temperatures were obtained by real-time measurement using a thermocouple.

## Data Availability

The data presented in this study are available on request from the corresponding author. The data are not publicly available due to privacy of study participants.

## Supplementary Materials

The following supporting information can be downloaded at: https://www.mdpi.com/article/10.3390/molecules27196197/s1, Figure S1: Pictures of chocolate sample before and after ashing; Table S1: Operating parameters of the GF-AAS for Cd analysis by microwave digestion; Table S2: Operating parameters of the microwave digestion for the sample of chocolate.

## Sample Availability

Samples are purchased from local supermarket and online shopping by the authors.
