# Supplementary material for: Fast Detection of Cadmium in Chocolate by Solid Sampling Electrothermal Vaporization Atomic Absorption Spectrometry and Its Application on Dietary Exposure Risk Assessment
Source: Molecules. 2022 Sep 21;27(19):6197. doi: 10.3390/molecules27196197 (PMC9572000; doi:10.3390/molecules27196197)
Supplement: Supplementary file 1 [file molecules-27-06197-s001.zip › molecules-1889724-supplementary.pdf]

Supporting Materials for

Fast Detection of Cadmium in Chocolate by Solid Sampling Electrothermal Vaporization Atomic Absorption Spectrometry and Its Application on Dietary Exposure Risk Assessment

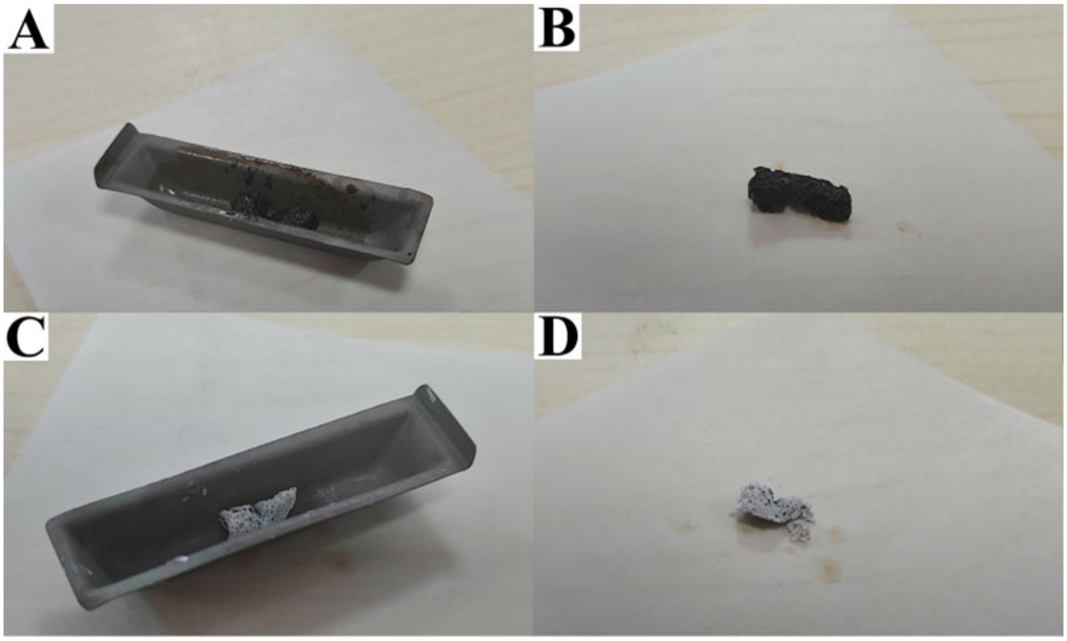

Figure S1. Pictures of chocolate sample before and after ashing.

Herein, 0.1 g chocolate sample was employed. A: The sample picture in nickel boat before ashing; B: The sample picture before ashing; C: The sample picture in nickel boat after ashing; D: The sample picture after ashing.

Table S1. Operating parameters of the GF-AAS for Cd analysis by microwave digestion.

Program	Target temperature (°C)	Heating rate (°C/s)	Hold time (s)
Dehydration	105	5	20
Pyrolysis	450	300	20
Atomization	1600	1500	3
Clean	1800	500	3

Table S2. Operating parameters of the microwave digestion for chocolate sample.

Program	Heating time (min)	Digestion temperature (°C)	Digestion pressure (psi)	Hold time (min)
Pre-digestion	5	130	350	5
Microwave digestion	5	180	350	10
